# Efficient end-to-end simulation of time-dependent coherent X-ray scattering experiments

**DOI:** 10.1107/S1600577524001267

**Published:** 2024-03-22

**Authors:** Himanshu Goel, Oleg Chubar, Ruizi Li, Lutz Wiegart, Max Rakitin, Andrei Fluerasu

**Affiliations:** a Electrical and Computer Engineering, Stony Brook University, 100 Nicolls Road, Stony Brook, NY 11794, USA; b Brookhaven National Laboratory, Upton, NY 11973, USA; ESRF – The European Synchrotron, France

**Keywords:** *Synchrotron Radiation Workshop*, X-ray photon correlation spectroscopy, coherent-mode decomposition, GPU acceleration

## Abstract

Physical optics simulations for beamlines and experiments allow users to test experiment feasibility and optimize beamline settings ahead of beam time in order to optimize valuable beam time at synchrotron light sources like NSLS-II. Further, such simulations also help to develop and test experimental data processing methods and software in advance. The *Synchrotron Radiation Workshop* (*SRW*) software package supports such complex simulations. We demonstrate how recent developments in *SRW* significantly improve the efficiency of physical optics simulations, such as end-to-end simulations of time-dependent X-ray photon correlation spectroscopy experiments with partially coherent undulator radiation (UR). The molecular dynamics simulation code *LAMMPS* was chosen to model the sample: a solution of silica nanoparticles in water at room temperature. Real-space distributions of nanoparticles produced by *LAMMPS* were imported into *SRW* and used to simulate scattering patterns of partially coherent hard X-ray UR from such a sample at the detector. The partially coherent UR illuminating the sample can be represented by a set of orthogonal coherent modes obtained by simulation of emission and propagation of this radiation through the coherent hard X-ray (CHX) scattering beamline followed by a coherent-mode decomposition. GPU acceleration is added for several key functions of *SRW* used in propagation from sample to detector, further improving the speed of the calculations. The accuracy of this simulation is benchmarked by comparison with experimental data.

## Abstract 2

End-to-end simulation of time-dependent partially coherent X-ray scattering experiments is demonstrated with the *SRW* code. A coherent-mode decomposition and GPU acceleration recently implemented in the code make such simulations feasible and efficient for a typical XPCS experiment at a storage ring light source. This enables more detailed tests and optimization of experimental configurations and data processing prior to beam time as well as understanding experimental data.

## 1. Introduction

The ability to study materials at the nano- and mesoscale is very important for the progress of modern science and technology. Various experimental techniques, such as high-resolution microscopy, coherent diffractive imaging and ptychography, as well as coherent X-ray scattering and other techniques are used for these purposes at modern high-brightness and high-coherence synchrotron light sources. Among the coherent X-ray scattering based techniques, X-ray photon correlation spectroscopy (XPCS) is of special interest owing to its ability to provide insights into the dynamics of samples which are difficult to obtain using other techniques (Nogales & Fluerasu, 2016 ▸).

The interest in utilizing the capabilities of synchrotron light sources for a large variety of experiments with X-rays is currently very high, making beam time a very precious resource. The ability to perform detailed simulations which have sufficient correspondence with experimental data can therefore be especially useful for improving the experimental throughput of light sources by reducing the amount of time spent on feasibility tests, selection of optimal beamline settings for given experiments, and making it easier to develop and test experimental data processing algorithms. This can also improve our understanding of experimental data by making it possible to compare them against several different models, which can be more precisely controlled than a real experimental environment. Simulation of experiments is also useful at the beamline design stage, where simulations are used to optimize beamline layouts to better meet the requirements of experiments to be supported at the beamline.

In this work, we focus specifically on demonstrating the simulation of XPCS experiments. A complete end-to-end simulation involving the XPCS analysis of Brownian diffusion of silica nanoparticles suspended in water is performed to match measurements made at the Coherent Hard X-ray (CHX) beamline at NSLS-II. We discuss the ability of recent additions to the *SRW* software package to closely match the results of time-dependent experimental measurements with very numerically efficient simulations (Chubar & Elleaume, 1998 ▸). Additionally, we demonstrate the application of recent developments in coherent-mode decomposition (CMD) methods towards efficiently accounting for partial coherence in simulations (Li & Chubar, 2022*a* ▸,*b* ▸).

We also show large improvements in calculation speed due to shifting some important steps of the simulation to graphics processing units (GPUs) that implement compute-unified device architecture (CUDA) support – a popular parallel computing platform that includes a programming language based on C++ which is used to write code for NVIDIA GPUs (Vingelmann & Fitzek, 2022 ▸). These developments, when used together, are key to making complex simulations tractable. Without the large improvements in calculation efficiency attributed to CMD methods and GPU utilization, such time-dependent simulations that emulate experimental conditions would be impractical, taking a long time to run even on large supercomputers. Although we demonstrate an XPCS experiment, these developments are applicable to significantly improving the fidelity and correspondence with experimental conditions for simulations of many other kinds of X-ray techniques, such as performing full-scale ptychography experiments where the effect of partial coherence of the beam on the reconstruction is considered.

Though previous works have discussed aspects of the coherent-mode decomposition and GPU accelerated computation in *SRW* (Goel *et al.*, 2022 ▸, 2023 ▸; Li & Chubar, 2022*a* ▸), this work demonstrates a qualitative leap in the detail of time-dependent partially coherent X-ray scattering experiments achieved by combining both and by performing a comparison with experimental data.

## 2. Coherent X-ray scattering experiments at a light source

Although large-emittance first- and second-generation light sources produced low-coherence X-ray beams, experimental techniques exploiting coherence started to be developed at second-generation sources (Sutton *et al.*, 1991 ▸; Dierker *et al.*, 1995 ▸). The considerably higher brightness and coherence of the third- and fourth-generation light sources allowed for maturity and led to further proliferation of coherence exploiting techniques. However, while the coherence of these light sources is significantly improved, the X-ray radiation beams produced are still only partially coherent, requiring the coherent flux for experiments to be ‘filtered out’ from the total emitted flux. In these modern light sources, a beam with high coherent flux is obtained using undulators. The undulator radiation (UR) beam is monochromated at a required photon energy (to obtain the necessary degree of longitudinal coherence), and a transversely coherent portion of this beam is ‘filtered out’ and transported to an experimental sample by X-ray beamline optics. The Coherent Hard X-ray (CHX) beamline at NSLS-II is designed and operates following this concept (Fluerasu *et al.*, 2011 ▸; BNL, 2016 ▸). The optical design of the CHX beamline allows for a controllable trade-off between the degree of X-ray coherence and flux at the sample. Thus, accurately simulating coherent X-ray scattering experiments at CHX still requires the consideration of partial coherence of the radiation illuminating the experimental sample.

Another crucial factor in increasing the quality of data in coherent X-ray scattering experiments is the significant improvement in detectors, which have progressed towards very high dynamic range per pixel – offering single-photon sensitivity – high pixel counts and small sampling times (Hill *et al.*, 2020 ▸). Further iterative improvements in these properties have also made it possible to study samples with faster dynamics. For the simulation of such time-resolved experiments, very high numerical efficiency of computation is required.

### 2.1. XPCS data processing

A typical XPCS experiment involves exposing a sample to a partially coherent X-ray beam and collecting the time-resolved scattering pattern. Statistical properties of the sample like the diffusion coefficient can then be determined by analyzing the changes in the speckle pattern as a function of time. For this, it is useful to examine multiple orders of correlation functions and their relation to the sample dynamics.

We start by investigating the two-time intensity–intensity autocorrelation function defined as

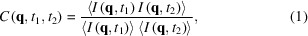

where *I*(*q*, *t*_1,2_) is the speckle intensity at wavevector **q** at times *t*_1,2_ and 〈…〉 represents the ensemble averaging operation (Madsen *et al.*, 2010 ▸), *i.e.* averaging over all detector pixels with the same **q**.

For equilibrium dynamics, the correlation between speckle pattern at a given wavevector only depends on the lag time τ = |*t*_2_ − *t*_1_| and the dynamics can be described by the one-time intensity–intensity auto-correlation function

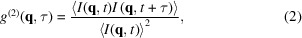

where the average 〈…〉 is taken not only over pixels with the same wavevector but also over time *t*.

The one-time intensity–intensity correlation function *g* ^(2)^ is related to the intermediate scattering function *g* ^(1)^ by the Siegert relation (Siegert, 1943 ▸),

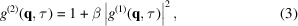

where β represents the speckle visibility.

Assuming that the Brownian motion of the particles is uncorrelated, the intermediate scattering function is only dependent on the wavevector **q**, representing the scattering angle relative to the beam center and the lag time τ,

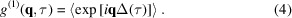

Assuming that the nanoparticles are sparsely distributed – as is this case – the mean squared displacement Δ(τ) is simply related to the diffusion coefficient *D*_*t*_ as

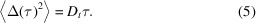

Using the Stokes–Einstein equation (Einstein, 1905 ▸) for the diffusion coefficient, we have

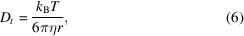

where *k*_B_ is the Boltzmann constant, *T* is the temperature, *r* is the particle radius and η is the viscosity. Thus, the expected form of the intermediate scattering function is

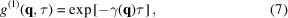

such that the relaxation rate is related to the diffusion coefficient by γ(**q**) = **q** ^2^ *D*_*t*_. Thus, for Brownian motion, the diffusion coefficient can be obtained from the intensity–intensity correlation function by curve fitting to

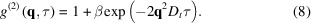

In this work, the XPCS data processing for the simulation was implemented using the implementations of these one-time and two-time correlation functions provided in the *scikit-beam* library (Abeykoon *et al.*, 2016 ▸).

## 3. Simulations of coherent scattering experiments

End-to-end simulations of time-dependent coherent X-ray scattering experiments allow for more efficient use of light sources by making it possible for users to test the feasibility of their experiment at an existing beamline and optimize the beamline settings, as well as develop new types of experiments and even suggest new beamlines for future experiments. For example, in an XPCS experiment carried out at a given beamline, users can predetermine whether the planned experiment would be sensitive to the dynamic phenomena taking place in specimens of scientific interest by estimating the necessary exposure times, scattering angles and image counts. In addition, end-to-end simulations can help to improve our understanding of complex dynamic phenomena beyond simple diffusion.

For best accuracy, the entire optical pathway of X-rays in a beamline needs to be simulated, starting from the synchrotron radiation source all the way to the detector. Luckily, most of the physical processes taking place at the emission of X-rays in insertion devices of light sources, their propagation through a beamline and even elastic interaction of the X-rays with the sample in scattering experiments can be accurately simulated in the framework of electrodynamics. For example, retarded potentials-based methods can be used for accurate near-field calculation of partially coherent UR by relativistic electrons moving in a storage ring, and the physical optics methods can be used for the simulation of propagation of this radiation to the sample, its interaction with the sample resulting in (coherent) scattering and propagation of the scattered radiation to the detector. For the efficiency of the physical optics calculations, Fourier optics and compatible methods can be used (Chubar & Elleaume, 1998 ▸).

Next, at the sample position, the cross spectral density of the partially coherent propagated radiation can be accumulated, and its coherent-mode decomposition can be computed for compact and, at the same time, highly accurate representation of this radiation in the simulation of the scattering experiments. This calculation is performed after propagating to the sample position because in the XPCS experiment all preceding optical elements of the beamline remain the same. Thus, this is the farthest point in the beamline at which the mode decomposition result can be shared. The radiation in the form of coherent modes is then propagated through the sample as defined by the spatial maps of the refractive index decrement and transmission length parameters of the materials involved. Finally, the radiation is propagated through free space to the detector. The finite spatial resolution of the detector is simulated by binning the electric field intensity based on detector pixel area. For a dynamic sample, *i.e.* when the spatial map of its refractive index has a time dependence, repeated propagation leads to a time series of speckle patterns that can then be processed via techniques such as XPCS.

Currently, the *SRW* framework is based on wavefront propagation with the paraxial approximation. This is sufficient for simulations in the SAXS regime, as presented in this work; however, simulations in the WAXS regime may need the consideration of dynamical X-ray diffraction effects, which are currently not used in this framework.

### 3.1. Coherent-mode decomposition to represent source and beamline

Coherent-mode decomposition methods were developed to simplify handling of the partially coherent characteristics of radiation by representing them as a sum of several fully orthonormal coherent modes. The number of modes needed was dependent on the degree of coherence of the radiation and the desired precision of the representation (Wolf, 1982 ▸). Typically, a very dense 4D grid of points is required to accurately sample the radiation, making these methods extremely demanding in terms of both simulation time and memory consumption. Recent developments have made it possible to reduce the required grid size and density by several orders of magnitude by subtracting quadratic phase terms (Li & Chubar, 2022*b* ▸). This optimized CMD method has been implemented in the *SRW* package and significantly reduces the computational complexity of simulations of partially coherent scattering processes.

**Figure 1:**
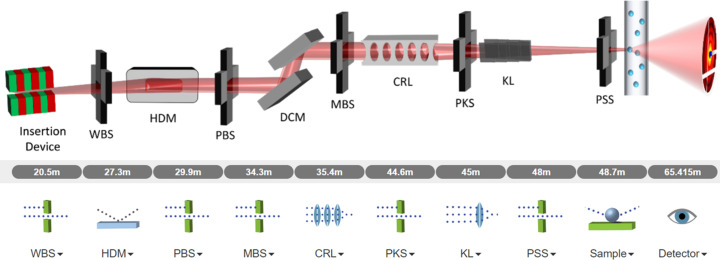
Optical layout of the CHX beamline for XPCS experiments (top) with the corresponding simplified *Sirepo* pictogram (bottom).

For this simulation, the virtual CHX beamline was configured – via the *Sirepo *web-based interface – to match the configuration of the beamline used in the experimental measurements, resulting in the bottom pictogram in Fig. 1 ▸ (Rakitin *et al.*, 2018 ▸). The insertion device was an in-vacuum undulator with a 20 mm magnetic period, configured to be at 9.65 keV at the fifth harmonic. The white beam slits (WBS), pink beam slits (PBS), mono beam slits (MBS), pre-kinoform slits (PKS) and pre-sample slits (PSS) were set to 0.6 mm × 1 mm, 0.6 mm × 1.2 mm, 0.1 mm × 0.4 mm, 0.2 mm × 0.1 mm and 0.029 mm × 0.029 mm, respectively. The stack of beryllium compound refractive lenses (CRLs) consisted of seven lenses with a 0.5 mm radius of curvature and one lens with a 1.5 mm radius of curvature. The kinoform lens was designed to focus the 9.65 keV X-rays just upstream of the sample position (Sandy *et al.*, 2010 ▸). The beam size is determined by the WBS and MBS, while the other slits clean parasitic scattering, which resulted in a beam size of ∼10 µm × 10 µm at the sample position for the given configuration.

Additionally, as seen in Fig. 1 ▸, a double-crystal Si(111) monochromator (DCM) would also be present between the PBS and MBS, but it was removed in the *Sirepo* model to simplify the simulation. Experiences from other beamlines suggest that, in SAXS experiments, the effect of the longitudinal coherence of the beam can be ignored due to small path length differences (Wiegart *et al.*, 2019 ▸); therefore, we ignore the effects of finite bandwidth for the purposes of the simulation and assume the monochromator to be ideal. However, our framework does have propagators for perfect-crystal monochromators if required.

After configuring the virtual beamline, the cross-spectral density was accumulated by propagating 10^5^ macro-electrons from the source to the sample position. The mode decomposition was computed from this accumulated cross-spectral density into 50 orthonormal coherent modes, the accumulated intensity of which corresponds to the characteristics of the partially coherent wavefront at the sample position. This allows all timesteps of the simulation to share these same coherent modes, making CMD orders of magnitude more efficient than performing an *ab initio* direct intensity averaging calculation for each timestep.

**Figure 2:**
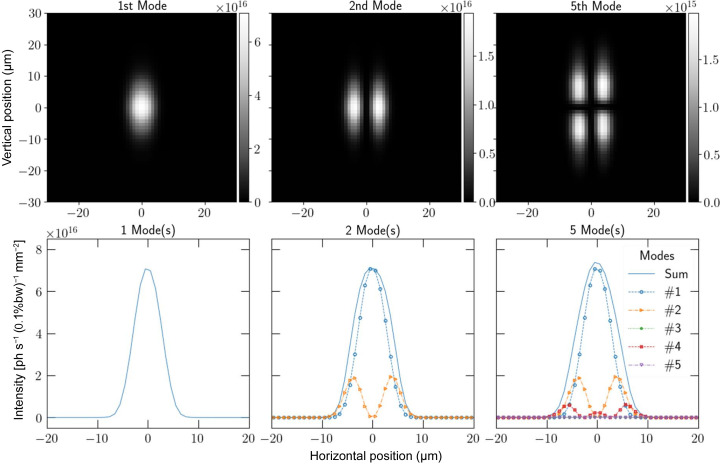
2D intensity of up to five coherent modes of the CHX beamline at the sample position with a horizontal cut (at vertical position zero) of their progressively accumulated intensity.

**Figure 3:**
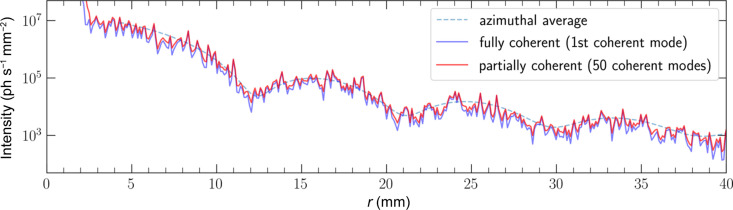
Diagonal cut of the speckle pattern obtained from the first frame of the molecular dynamics simulation for a colloidal suspension of silica nanoparticles.

The CHX beamline is configured to filter out a large portion of the incoherent radiation produced by the insertion device, resulting in a beam with high transverse coherence at the sample position. This design is reflected in the intensities of the coherent modes, as shown in Fig. 2 ▸. Higher order modes have much smaller intensity, making the propagation of partially coherent radiation significantly more efficient than performing an *ab initio* direct intensity averaging calculation by changing the problem of propagating several thousand macro-electrons per frame to only propagating several dozen coherent modes per frame. The contribution of coherent modes of an order greater than ∼50 became of too small of a relative magnitude to significantly change the resulting speckle pattern, with the 50th mode having a peak intensity which is 0.04% of the peak intensity of the first mode. This means that the first 50 modes are sufficient for accounting for most effects of partial coherence in this simulation. The difference in the scattering intensity distribution for only the first coherent mode compared with the accumulation of the first 50 coherent modes for the first frame of the simulation data is shown in Fig. 3 ▸, showing the ‘smoothing’ of the speckles indicative of the effects of partial coherence.

The property of needing fewer coherent modes as the transverse coherence of the beam increases can also be utilized to progressively refine the beamline configuration using coherent modes pre-calculated at the insertion device, then propagating the modes through to the sample position and recomputing the CMD. This would allow the optimization to be easily applied to obtain the smallest possible number of coherent modes for any beamline configuration, before finally performing scattering calculations with those modes. This also enables more efficient exploration of the costs and benefits of adjusting parts of the beamline configuration – such as slit sizes, controlling the trade-offs between the degree of coherence, flux and exposure time – on the results, by progressively computing the coherent modes and performing the simulation with those coherent modes.

### 3.2. GPU acceleration

The emergence and widespread accessibility of massive parallel computation platforms in the form of GPUs opens new avenues for further acceleration of physical optics simulations. GPU usage is also important for making effective use of next-generation GPU-focused supercomputers like the NERSC Perlmutter, where 60 PFLOPS of the system’s 64 PFLOPS performance rating are derived from its GPUs (NERSC, 2021 ▸).

**Figure 4:**
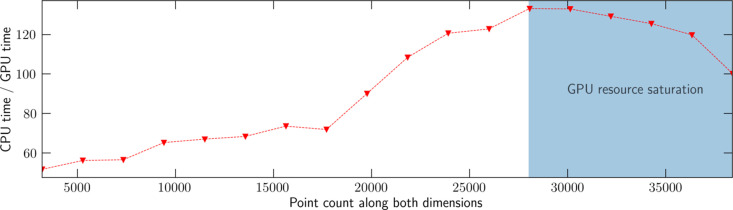
CPU time/GPU time versus point count of the simulation grid along both dimensions.

As an exploratory step, several key components of the *SRW* radiation propagation codes were re-implemented in CUDA. CUDA implementations were mainly added for the optical element propagators responsible for simulating propagation of a wavefront through free space and through a 2D sample. When comparing performance measured on an AMD Ryzen 3900X CPU against an NVIDIA RTX 3090 GPU, this resulted in the several orders of magnitude speed improvement compared with the CPU shown in Fig. 4 ▸, varying depending on the point count used for the propagation. After simulation grid sizes exceed ∼(2.8 × 10^4^)^2^ points, slowdowns are encountered due to saturation of several GPU resources such as computational parallelism and memory bandwidth, combined with increasing overhead of factors such as the amount of memory available on the GPU and the time taken to transfer data to and from the GPU to the CPU. The conversion was done such that the use of GPUs is completely transparent to the *SRW* simulation script, making it very simple to accelerate existing simulations. The library can also revert to the CPU versions of the propagators on request or if no GPU is available.

The optical element propagators involved in the sample-to-detector propagation were reimplemented in CUDA, corresponding to transmission through a 2D sample element and then propagation through free space. The transmission propagator implements

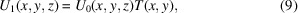

where *U*_0_(*x*, *y*) are the initial electric field components and *T*(*x*, *y*) are the 2D complex transmission functions representing the absorption and phase shift caused by the sample at the *x*, *y* position attributed to the thickness of the sample at that point and the material the sample is composed of.

Similarly, the free space propagator was reimplemented in CUDA as a Fresnel propagator under the paraxial approximation

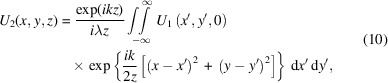

where *k* = 2π/λ, λ is the wavelength of the radiation and *z* is the distance to be propagated, which in this case would be the sample-to-detector distance. This equation can be reduced to a 2D Fourier transform, simplifying the implementation significantly by allowing for the use of existing optimized libraries for the calculation of Fourier transforms. In the CUDA implementation, the cuFFT library is used to perform these.

Additionally, to utilize GPU acceleration most effectively in *SRW* while reducing the complexity of managing memory between the CPU and GPU(s), a memory tracking system was added to opportunistically transfer data to and from the GPU VRAM buffer depending on where the data are needed. This helps compensate for the limited size of GPU memory compared with the large amounts of CPU memory available on machines intended for high performance computing. It also allows the components of *SRW* which have not received CUDA implementations to be used alongside GPU-enabled code by ensuring that GPU calculations have finished before the CPU-only code is executed. To reduce the overhead of data transfers, the system also tracks the data dependencies between the steps of the simulation and uses this information to utilize the ability of recent GPUs to perform memory transfers and calculations simultaneously. This design also helps to make the process of re-implementing simpler components of *SRW* into CUDA much faster and creates new opportunities for the development of new hybrid optical propagators which combine both CPU and GPU computation. This tracking system is also designed to be largely independent of *SRW* itself, allowing for it to be employed in porting other similar codes.

### 3.3. High-accuracy simulation of samples

**Figure 5:**
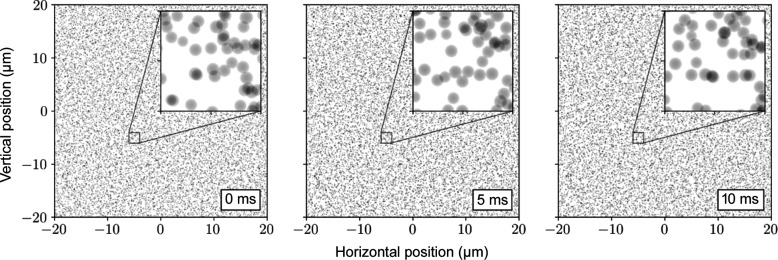
Optical path length differences representing 4 µm-thick slices of the molecular dynamics simulation volume for a colloidal suspension of silica nanoparticles in water taken at different timesteps of the simulation.

To simulate realistic experiments, the ability to import generic sample data is required. This can be used, for example, to import simulation results from molecular dynamics packages as samples against which to calculate scattering. For this, the ability to import simulation data was added through a simple text-based format consisting of a list of 3D objects with their real space positions, shape and shape-specific properties. The format was designed with the goal of making it easy to import data from any source. Currently, only sphere definitions are supported but the format is designed to be easily extended to support other shapes. These data are rendered into optical path differences as shown in Fig. 5 ▸, with the optical path length difference depending on the thickness of the object, and the refractive index decrement and absorption length of the material the samples are made of at the X-ray energy of the experiment (Chubar *et al.*, 2020 ▸).

In this case, the *LAMMPS* molecular dynamics simulation package (Thompson *et al.*, 2022 ▸) was used to simulate spherical silica nanoparticles with an average radius of 125 nm suspended in water at room temperature with 1.6% size polydispersity. The package was configured to output a list of sphere spatial positions and radii for each timestep, which was easily converted to the format designed for *SRW*. A simulation volume of 40 µm × 40 µm × 40 µm was used with a 0.02% volume fraction of nanoparticles. A 4 µm-thick slice of the data was imported into *SRW*.

The Brownian motion of particles is described by the diffusion coefficient calculated from the Stokes–Einstein diffusion equation [equation (6)]. The viscosity of water at 297.15 K was taken to be 0.929 × 10^−3^ Pa s from the experimental data. A simulation timestep of 100 µs was used, with an initial 40 ms of equilibration and 1 s of data collection from a uniform grid with periodic boundary conditions. A 10 ms slice of the data was used for the XPCS analysis. The mean-squared displacement of the simulation was used to verify the correctness of the simulation.

### 3.4. Total improvement

By combining CMD methods with GPU acceleration, a greater than three orders of magnitude net speedup was obtained compared with *ab initio* calculations, bringing calculations that would have taken many days to perform on a supercomputer into the realm of a one-time cost of hours on a supercomputer to calculate the CMD once and then dozens of minutes to calculate all the scattering patterns on a typical GPU workstation. This represents a large increase in accuracy and computational efficiency of the simulation for the same calculation duration. A large reduction in peak memory consumption was also achieved, making it possible to perform much more detailed simulations which account for partial coherence of real light sources. The speed of the GPU accelerated calculations and the ability to reuse the coherent modes makes rapid iteration of samples practical. Simpler simulations also benefit, with the possibility of using higher amounts of parallelism for the calculations. Furthermore, these improvements allow *SRW* to take advantage of any GPU which supports CUDA. Given the increasing popularity of such in both supercomputers (Khan *et al.*, 2021 ▸) and personal computers, these developments set up the code for further large improvements in efficiency, accuracy and simulation complexity across the board as additional functionality is made to be GPU-accelerated.

## 4. Comparison with experimental data

The accuracy our simulations can achieve was tested by tuning the simulation to be comparable with an experimental measurement of the same kind of sample. We found that close correspondence was achieved by accounting for mirror reflectivity and monochromator bandwidth based on the parameters provided by Wiegart *et al.* (2017 ▸).

The 50 coherent modes were propagated through a 4 µm-thick slice of the simulation volume – approximated to be 2D, calculating single scattering of the radiation incident on the sample. The detector was set to be 16.715 m from the sample and was simulated with a pixel size of 75 µm × 75 µm, matching the Dectris Eiger-X 500k detector used for this measurement.

A notable difference between simulation and the experiment was that, while the simulations propagate EM-fields, experimental data are collected by photon counting. Thus, the simulation obtains scattering intensities without the effect of the low photon counts involved in the experiment, resulting in the simulated scattering patterns being more precise than those collected in the experiment.

**Figure 6:**
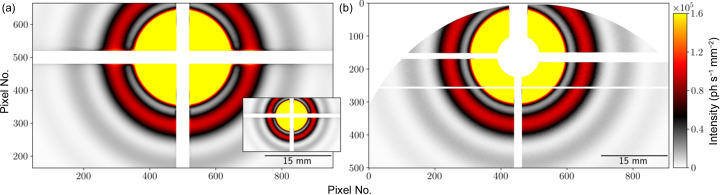
Averaged speckle patterns in linear scale from averaging over 10^4^ frames (10^2^ frames in inlay) from simulation (left) and experiment (right).

Fig. 6 ▸ shows the averaged scattering patterns: Fig. 6 ▸(*a*) shows the average of all 10^4^ frames and an inlay of the average of 10^2^ frames, and Fig. 6 ▸(*b*) ▸ shows the average of 10^4^ of experimental data. With only 10^2^ frames, the pattern lacks enough samples to average out all the speckles. The intensity thresholds have been set to emphasize the rings which result from the particles in the sample being spherical with low polydispersity. The intensity at the center of the beam is several orders of magnitude higher than at the rings, thus requiring the upper range to be clipped to make the rings easily visible.

**Figure 7:**
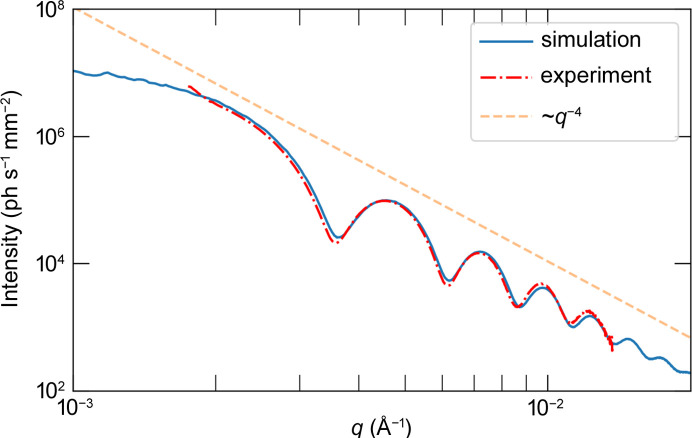
Comparison of circularly averaged SAXS curve from simulated and experimental data.

The SAXS curve was obtained by taking the azimuthal average around the beam center over the average of all frames of data. In the SAXS curve, the ‘sharpness’ of the valleys is representative of the polydispersity of the particles and their distribution in the sample. This curve is very sensitive to minor variations in particle size and distribution, which is reflected in the deviations of the simulated curve from the experimental curve in Fig. 7 ▸.

**Figure 8:**
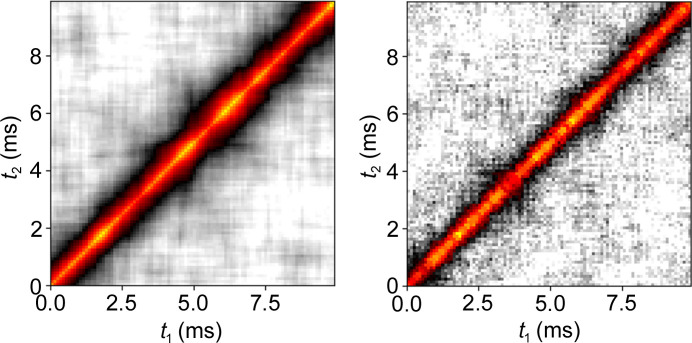
Two-time correlation function at **q** = 0.00172 Å^−1^ for simulation (left) and experiment (right).

**Figure 9:**
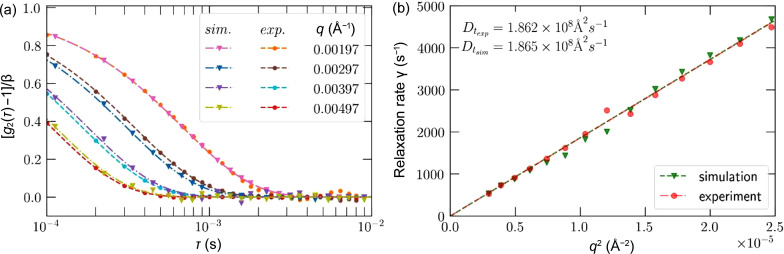
(*a*) One-time correlation functions for different **q**-values for both simulation and experiment. (*b*) Diffusion coefficients obtained from the relaxation rates of the one-time correlations.

For the XPCS analysis, the two-time and one-time correlation functions – equations (1) and (2), respectively – were calculated for experimental and simulated data for the same wavevectors and plotted along with fits to the one-time correlation. The two-time correlations shown in Fig. 8 ▸ indicate the correlation of each timestep of the data with all other timesteps of the corresponding dataset. Highly correlated contours parallel to the diagonal were observed with a sharp dropoff, showing that correlations are dependent only on the time difference, indicating equilibrium dynamics as would be expected for this sample. The correlation time – seen as the width of the diagonal – was also comparable between simulation and experiment. From the two-time correlations, the one-time correlation was calculated by curve fitting to two-time correlation points for several **q**-values, with the points indicated by the markers in Fig. 9 ▸(*a*) and the corresponding curves being the result of curve fitting to obtain the relaxation rates.

The diffusion coefficients were obtained by performing a linear fit of the relaxation rate γ versus wavevector **q** ^2^ as shown in Fig. 9 ▸(*b*), demonstrating that the experimental and simulated diffusion coefficients for both cases are within <0.2%.

## 5. Conclusions

We have demonstrated a more than three orders of magnitude net simulation speedup for end-to-end partially coherent scattering experiments by combining CMD methods and GPU acceleration, expanding the range of partially coherent scattering simulations that can be performed. These improvements make detailed simulations of complex experiments possible, which was demonstrated through the example simulation of an XPCS experiment. Simulation results were shown to be in close agreement with both the parameters used to perform the molecular dynamics simulation and the results for a similar experiment performed at NSLS-II.

These results represent a big step towards connecting simulations of samples with their associated real-world experiments to make it easier to understand and interpret experimental data, which can significantly improve the ease-of-use and productivity of synchrotron light sources.

These developments can support many applications of significant use to light source users and beamline scientists, such as performing more precise studies on the feasibility of an experiment and the acceptability of the trade-off between coherence, flux and the exposure times needed for the sample. This combination also creates possibilities for detailed simulations of other kinds of experiments such as ptychographic imaging of samples.
